# Bilateral Asynchronous Displaced Olecranon Fractures in a Patient With Osteogenesis Imperfecta

**DOI:** 10.7759/cureus.23433

**Published:** 2022-03-23

**Authors:** Rachel A Thomas, William Hennrikus

**Affiliations:** 1 Orthopaedic Surgery, Penn State Health Milton S. Hershey Medical Center, Hershey, USA

**Keywords:** tension band wiring, elbow, pediatric, osteogenesis imperfecta, olecranon fracture

## Abstract

Olecranon fractures are uncommon in children. Children with osteogenesis imperfecta (OI) are at an increased risk of olecranon fractures. This is a report of a 12-year-old male patient with known osteogenesis imperfecta type 1 who sustained bilateral asynchronous olecranon metaphyseal avulsion fractures. He sustained a right olecranon avulsion fracture from a fall and underwent open reduction and internal fixation with two Steinman pins and a tension band wire. He was placed in a cast for a month. The Steinman pins were removed at three months. Six months after the first fracture, he sustained a left olecranon avulsion fracture while playing soccer. He underwent open reduction and internal fixation with two Steinman pins and tension band wiring. Hardware was removed at three months. He was returned to full activity due to his type 1 OI. Bilateral asynchronous avulsion fractures of the olecranon are rare, except in children with OI. In the current case, good functional recovery was obtained with tension band wiring.

## Introduction

Isolated olecranon fractures are uncommon injuries in children. They typically occur following a direct blow to the olecranon or a fall onto a flexed elbow. Children with osteogenesis imperfecta (OI) have an increased incidence of this injury and a high incidence of bilateral asynchronous olecranon fractures [1,2]. This injury is more commonly seen in type 1 OI, compared to other more severe forms of the condition [2-5]. Avulsion fracture of the olecranon is the most common fracture pattern in children with OI [1,4-6]. Displaced olecranon fractures are managed with open reduction and internal fixation (ORIF) [1,2,4,6]. In the case reported, a 12-year-old male with known type 1 OI sustained bilateral asynchronous displaced olecranon fractures. This study was approved by our institutional review board.

## Case presentation

A 12-year-old male with a history of osteogenesis imperfecta type 1 sustained bilateral asynchronous displaced olecranon fractures. The diagnosis of OI was made by a geneticist at age five. The first fracture occurred when the patient fell on the stairs, sustaining a right olecranon fracture. On the physical exam, his sclera was blue. His right elbow was swollen and ecchymosis was present over the olecranon.

The patient previously sustained fractures of the fingers, clavicle, and feet. All resulted from trauma and healed uneventfully. He takes vitamin D, independent of his OI. His baseline activity was full sports activity due to his minor form of OI.

**Figure 1:**
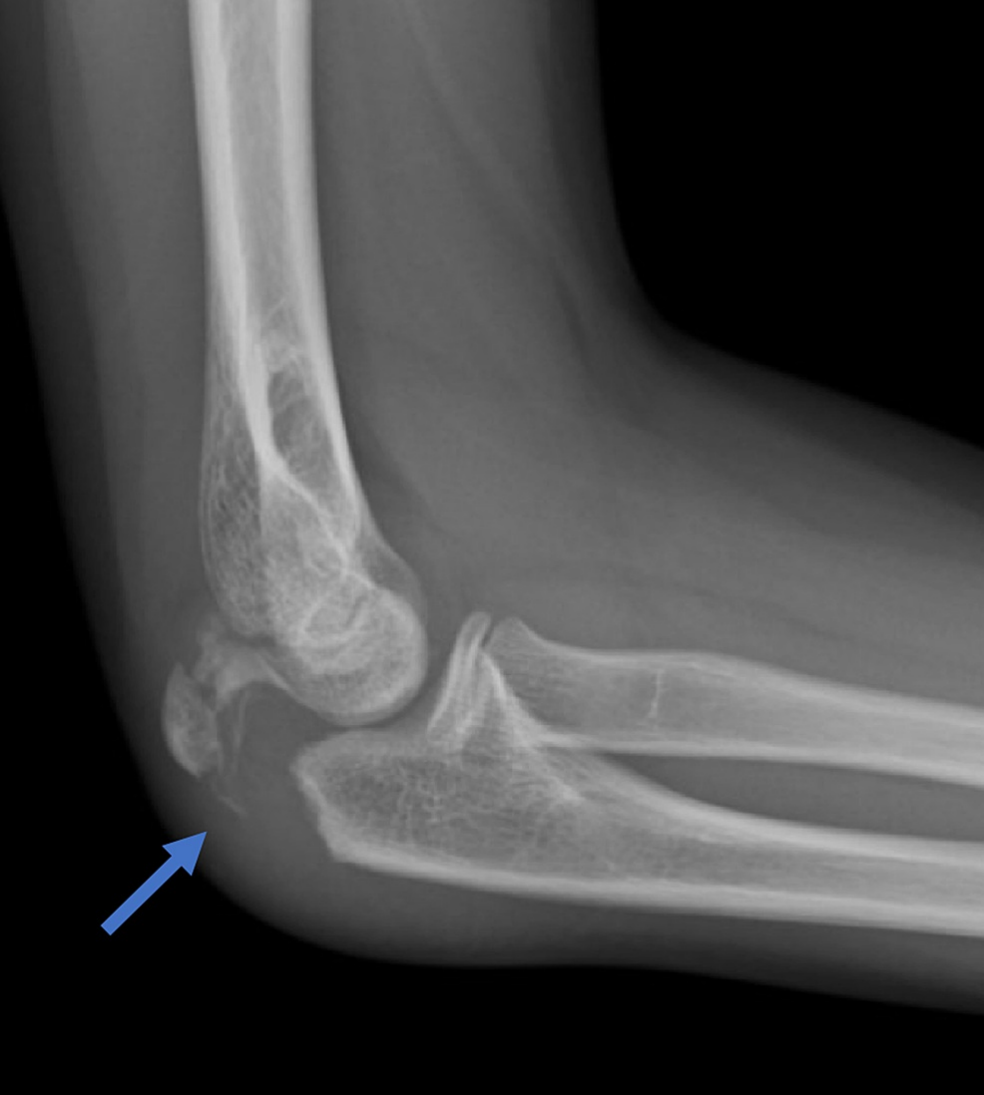
Lateral radiograph of the right elbow shows a displaced olecranon metaphyseal fracture (arrow).

**Figure 2:**
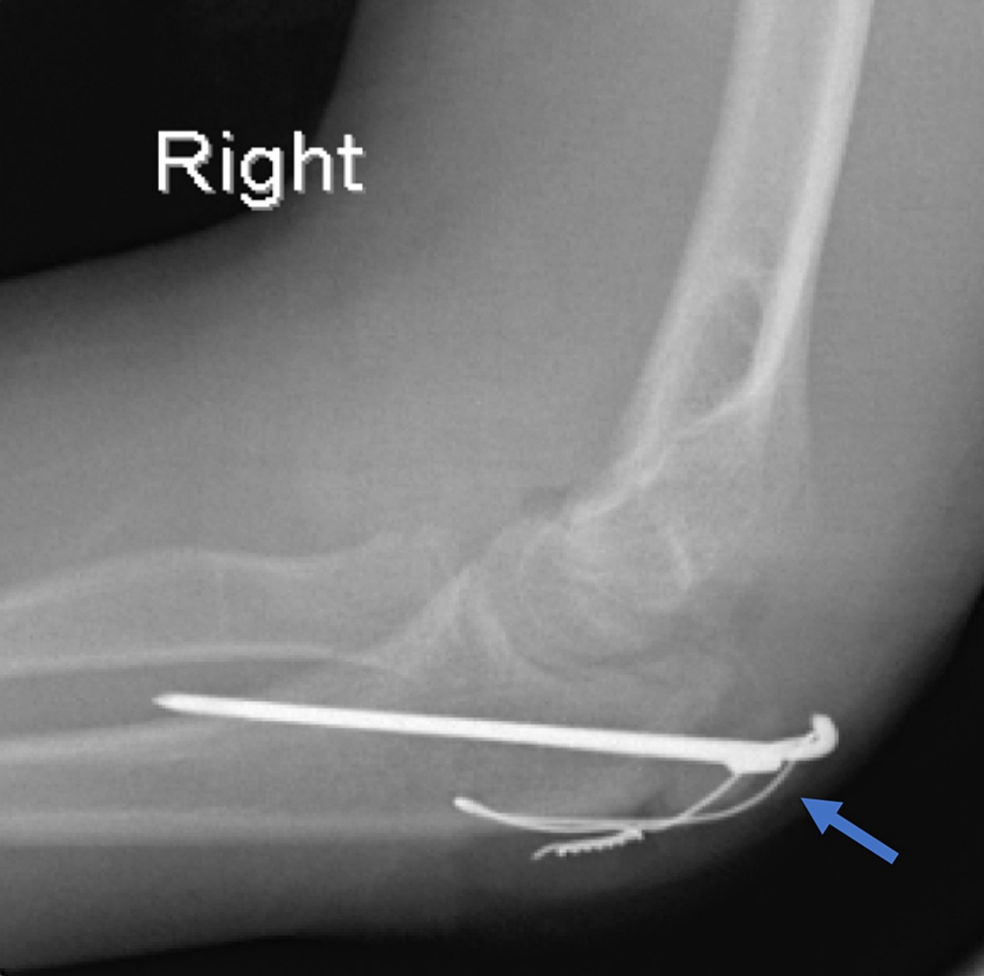
Lateral radiograph of the right elbow showing the tension band wire (arrow) technique with two 2 mm Steinman pins and an 18-gauge wire tension band.

Radiographs demonstrated an isolated displaced fracture of the olecranon metaphysis. The fracture was displaced 11 mm on the lateral radiograph of the right elbow (Figure 1). The patient underwent open reduction internal fixation (ORIF) with tension band wire (TBW) under general anesthesia (Figure 2). Post-operatively, the patient was treated in a well-padded long-arm cast. As a precaution, the cast was split. 

One-month post-operatively, the cast was removed. Once the cast was removed, we encouraged a range of motion. The patient was held out of sports for two months. At the two-month follow-up, the range of motion was -3 to 115 degrees, with a carrying angle of 8 degrees, supination of 80 degrees, and pronation of 80 degrees. Hardware was removed at three months. 

Six months after sustaining a right olecranon fracture, the patient sustained a second injury. The patient fell while playing soccer, sustaining a displaced left olecranon fracture. On presentation, he was neurovascular intact and had tenderness to palpation over the olecranon. Radiographs demonstrated an isolated displaced left olecranon fracture of the olecranon metaphysis. The fracture was displaced 15 mm on the lateral radiograph.

The patient underwent ORIF of the left olecranon TBW technique and was placed in a well-padded long-arm cast. The cast was split. One-month post-operatively, the cast was removed. Hardware was removed at three months. At the final follow-up, four months after the left elbow fracture, the patient demonstrated full range of motion and returned to full activity.

## Discussion

A case of asynchronous olecranon avulsion fractures in a patient with osteogenesis imperfecta. In children with OI, there is a higher incidence of isolated olecranon fractures than in the general population, most commonly occurring in OI type 1. Type 1 OI most commonly results from a premature stop codon of the COL1A1 allele [7]. The truncated RNA transcript is degraded, leading to decreased quantities of normal 1a collagen chains [7]. The sillence classification characterizes type 1 OI with mild bone fragility, blue sclera, and reduced collagen production [7]. Type 1 is subdivided into types 1A and 1B; 1A has normal tooth development and 1B has dentinogenesis imperfecta. Type 2 is autosomal recessive; the most common genetic mutation is a glycine substitution in COL1A1 or COL1A2, leading to a qualitative disorder of collagen [8]. Type 2 OI is lethal in the neonatal or perinatal period and is associated with the blue sclera. Type 3 is autosomal dominant and characterized by normal sclera, dentinogenesis imperfecta, short stature, progressive deformity, and a qualitative disorder of collagen [7,8]. Type 4 is autosomal dominant and characterized by the normal sclera, moderately short stature, dentinogenesis imperfecta, and a qualitative disorder of collagen [8]. Diaphyseal strength is decreased in OI due to decreased cortical bone thickness and increased vascular channels [3]. Fractures most commonly occur during adolescence, with increased activity and a period of rapid growth. Fractures are less common once individuals with OI reach adulthood [6].

Avulsion of the olecranon results from the contraction of the triceps when the elbow is flexed [1]. In children with OI, the fracture occurs in the metaphysis of the proximal ulna adjacent to the olecranon physis [5]. The weakness of the subchondral bone of the metaphysis directs the fracture into the metaphysis versus the physis. Since the fracture is directed into the metaphysis, sparing the physis, the risk of limb length discrepancy is low. The transversely oriented subchondral bone results in a thin layer of metaphyseal bone remaining attached to the apophyseal fragment [6]. As in the current case, the deforming force of the fracture proximally is the pull from the triceps insertion onto the olecranon and distally from the proximal pull of the brachialis origin [9].

Tension band wiring (TBW) is the preferred management of olecranon fractures in children with OI (Figure 2). Tension bands are used to convert displacing tensile forces into compressive forces of the triceps and brachialis across the fractures [9]. The distractive force of the triceps during active elbow flexion increases the compression [9]. Persiani et al. compared TBW with cannulated screw fixation [1]. They reported improved outcomes with TBW. TBW minimalizes immobilization and decreases the risk of physeal damage. The most common complication of tension band wire at the elbow is hardware discomfort requiring removal [2]. 

Patients with OI have a high risk of sustaining a contralateral olecranon fracture. Olecranon fractures occur in 8.1% of children with osteogenesis imperfecta [2]. Persiani et al. and Zionts et al. reported a 70% rate of contralateral olecranon fracture [1,5]. 5.7% of children with OI will sustain bilateral asynchronous olecranon fractures. Patients and their families should be counseled about the high rate of contralateral fracture. TBW is often symptomatic at the elbow and requires later removal [1,2,5].

In addition to olecranon fracture, cases of tibial tubercle avulsion fractures in patients with OI have been reported in the literature [10]. OI should be considered in children presenting with isolated tibial or olecranon avulsion fractures and other signs of OI, such as blue sclera and a history of fractures.

## Conclusions

Olecranon fractures are uncommon injuries in children, except in children with OI. Children with type 1 OI who sustain one olecranon avulsion fracture are at risk of a contralateral injury. Tension band wiring is the treatment of choice for olecranon fractures in children. The outcomes in this case were excellent.

## References

[1] Persiani P, Ranaldi FM, Graci J (2017). Isolated olecranon fractures in children affected by osteogenesis imperfecta type I treated with single screw or tension band wiring system: outcomes and pitfalls in relation to bone mineral density. Medicine (Baltimore).

[2] Tayne S, Smith PA (2019). Olecranon fractures in pediatric patients with osteogenesis imperfecta. J Pediatr Orthop.

[3] Peddada KV, Sullivan BT, Margalit A, Sponseller PD (2018). Fracture patterns differ between osteogenesis imperfecta and routine pediatric fractures. J Pediatr Orthop.

[4] Stott NS, Zionts LE (1993). Displaced fractures of the apophysis of the olecranon in children who have osteogenesis imperfecta. J Bone Joint Surg Am.

[5] Zionts LE, Moon CN (2002). Olecranon apophysis fractures in children with osteogenesis imperfecta revisited. J Pediatr Orthop.

[6] Ogden Ogden, JA JA (2000). Radius and ulna. Skeletal Injury in the Child, 3rd ed.

[7] Rowe DW (2002). Osteogenesis imperfecta. Principles of Bone Biology, 2nd ed.

[8] Fratzl-Zelman N, Misof BM, Roschger P, Klaushofer K (2015). Classification of osteogenesis imperfecta. Wien Med Wochenschr.

[9] Carter TH, Molyneux SG, Reid JT, White TO, Duckworth AD (2018). Tension-band wire fixation of olecranon fractures. JBJS Essent Surg Tech.

[10] Tamborlane JW, Lin DY, Denton JR (2004). Osteogenesis imperfecta presenting as simultaneous bilateral tibial tubercle avulsion fractures in a child: a case report. J Pediatr Orthop.

